# Altered Visual and Feet Proprioceptive Feedbacks during Quiet Standing Increase Postural Sway in Patients with Severe Knee Osteoarthritis

**DOI:** 10.1371/journal.pone.0071253

**Published:** 2013-08-22

**Authors:** Rogerio Pessoto Hirata, Tanja Schjødt Jørgensen, Sara Rosager, Lars Arendt-Nielsen, Henning Bliddal, Marius Henriksen, Thomas Graven-Nielsen

**Affiliations:** 1 Center for Sensory-Motor Interaction (SMI), Department of Health Science and Technology, Faculty of Medicine, Aalborg University, Aalborg, Denmark; 2 Clinical Motor Function Laboratory, The Parker Institute, Copenhagen University Hospital at Frederiksberg, Frederiksberg, Denmark; University of Ottawa, Canada

## Abstract

**Objective:**

The objective was to investigate how postural control in knee osteoarthritis (KOA) patients, with different structural severities and pain levels, is reorganized under different sensory conditions.

**Methods:**

Forty-two obese patients (BMI range from 30.1 to 48.7 kg*m^−2^, age range from 50 to 74 years) with KOA were evaluated. One minute of quiet standing was assessed on a force platform during 4 different sensory conditions, applied 3 times at random: Eyes open (EO) and eyes closed (EC) standing on firm and soft (foam) surfaces (EO-soft and EC-soft). Centre of pressure (Cop) standard deviation, speed, range and Cop mean position in both directions (anterior-posterior and medial-lateral) were extracted from the force platform data. Structural disease severity was assessed from semiflexed standing radiographs and graded by the Kellgren and Lawrence (KL) score. Pain intensity immediately before the measurements was assessed by numeric rating scale (range: 0–10).

**Results:**

The patients were divided into “less severe” (KL 1 and 2, n = 24) and “severe” (KL>2, n = 18) group. The CoP range in the medial-lateral direction was larger in the severe group when compared with the less severe group during EC-soft condition (*P<0.01*). Positive correlation between pain intensity and postural sway (range in medial-lateral direction) was found during EC condition, indicating that the higher the pain intensity, the less effective is the postural control applied to restore an equilibrium position while standing without visual information.

**Conclusion:**

The results support that: (i) the postural reorganization under manipulation of the different sensory information is worse in obese KOA patients with severe degeneration and/or high pain intensity when compared with less impaired patients, and (ii) higher pain intensity is related to worse body balance in obese KOA patients.

## Introduction

Knee osteoarthritis (KOA) is a progressive joint disease associated with pain in and around the knees [1], [2], impaired knee proprioception [3], balance impairments [4], [5], and increased risk of falling [6]. Pain per se has been associated with postural instability [7]–[11]. Interestingly, anaesthetizing the knee joint of KOA patients does not improve balance or knee proprioception, although it increases the maximum quadriceps strength one hour after a bupivacaine injection [12] supporting the notion that pain inhibits muscle activation [13] and torque [14], [15]. Thus, it seems that the mechanisms behind the impaired posture control in KOA involve on a complex interaction between the sensory information and the motor system controlling the body's center of mass [16], [17].

While standing, the body sways constantly, requiring continuous correction of its position to maintain stability. Three major sensory systems provide information for constructing an internal body model that represents the different body segments relative to the surrounding environment: (i) visual, (ii) vestibular, and (iii) somatosensory [18]. The information from these sensory systems is organized to generate muscle forces aiming to stabilize the body oscillations and maintain balance. In the presence of disease, some of these sensory systems might be impaired, requiring the ability to increase the contribution of unaffected areas and sensory systems for maintenance of postural orientation [19]. Such optimization might be achieved by reorganizing the gain of each sensory system to enhance balance in altered conditions [20], which might indicate a posture control reorganization. Such theory suggests that to maintain balance in a painful condition or disease, the central nervous system may increase the gain of sensory information from the non-affected areas, which provides correct information, and decrease the gain from impaired sensors. Aging processes impair the sensory integration in elderly people which compromises posture control enhancement [21], [22]. Additionally, when compared with healthy young subjects, healthy older individuals are less stable when two senses are manipulated at the same time [23], [24]. Obesity impairs balance in healthy subjects [25]–[28] and it is associated with the development [29], [30] and progression [31] of KOA. Furthermore, within the KOA patient population, there are indications that obesity is correlated with muscle weakness [32], which causes a lesser capacity to recover from balance disturbances [33]. This might explain why patients with higher body mass index (BMI) are more prone to fall compared with lower BMI patients [32]. In healthy subjects, Hirata *et* *al* [8] suggested that during a unilateral experimental knee-related pain condition, compensatory effects provided by the non-painful areas (non-affected side) was a possible explanation for the absence of postural stability changes. These results suggest that healthy subjects are capable of reweighting the sensory information in order to maintain balance. Additionally, previous studies showed that ageing [22] and loss of a particular sense, as observed in patients with vestibular loss [19] and blind subjects [34], does not prevent reweighting of sensory information while controlling balance. However, clinically it is still an open question if obese KOA patients are capable of reorganizing their sensory input, and how this capability is affected by the disease severity and pain intensity. Patients with chronic knee pain having decreased knee extensor and flexor muscles strength [35], a reorganization of the muscular response from the remaining healthy structures may generate alternatives for compensatory postural control. A better understanding of these mechanisms underlying the complex impaired behavior in obese KOA patients may benefit future rehabilitation protocols focusing on balance training to increase postural stability and reduce risk of falls.

The aim of the present study was to investigate: (i) posture control reorganization in obese KOA patients during different sensory conditions, (ii) the relationship between this reorganization and the KOA severity degree. We hypothesize that patients will have worst postural stability in conditions where the sensory information is altered and this impairment will be positively correlated with degeneration and/or pain intensity in these patients.

## Methods

### Subjects

The participants were recruited arbitrarily among patients at baseline of the CAROT study (influence of weight loss or exercise on cartilage in obese knee osteoarthritis patients (KOA) patients: a randomized controlled trial, CAROT (ClinicalTrials.gov identifier: NCT00655941) [36]. Forty-five (29 females) obese (BMI >30 kg*m^−2^) KOA patients were randomly included after giving their written informed consent. Eligibility criteria for the CAROT study were to be older than 50 years, with primary KOA diagnosed according to the American College of Rheumatology criteria [37] with clinical symptoms and radiographically or arthroscopically verified OA in one or both knees. Use of analgesics was allowed in the study, but not in a period of 24 h before tests. Recent studies indicate that experimental pain around the knee affects postural sway [8], [9] in healthy subjects. Assuming that pain also affects the postural sway in KOA patients, refraining from using analgesics 24 h before the data collection, allowed this study to address the effects of both pain and KL grade on the postural sway of KOA patients. Radiographic Kellgren and Lawrence (KL) score [grade 0 (normal) to grade IV] from the most affected knee was obtained. Although the KL grade 1 is generally considered to be questionable and often ignored in KOA studies, recent data suggest that even in early stages of the disease, patients with low KL grade (1 and 2) present significant pathological changes in most of the knee-related structures such as cartilage and menisci [38]. Therefore, the present study included patients with KL score from 1 to 4.

### Ethics Statement

The study was approved by the local ethical committees of the municipalities of Frederiksberg in Copenhagen (H-B-2007-088) and conducted in accordance with the Helsinki Declaration.

### Protocol

The patients were asked to stand as quiet as possible during one minute on a force platform measuring the ground reaction forces and moments produced by their postural sway. Four different experimental conditions were applied 3 times randomly to evaluate quiet standing balance: (i) Eyes open with firm surface, (ii) eyes closed with firm surface, (iii) eyes open with soft foam surface, and (iv) eyes closed with foam surface.

### Centre of pressure

The subjects stood barefoot and adopted a standard position with their feet comfortably positioned side-by-side (about shoulder width apart) and arms positioned along of the body, while standing on the force platform. The position of the feet was marked to ensure that the subjects always used the same position over all trials. The subjects were asked to focus on a fixation point (dark blue circle, 15 cm diameter) placed on a white wall 6 m in front of them. During the foam surface condition, the subjects stood on a 45.7×45.7×12.7 cm (length, width, height) foam (approx. density 60 kg*m^−3^) (NeuroCom®, USA) placed on top of the force platform. Line grids on the foam were used to mark the patient foot position in other to match with the position used during the firm surface condition.

The force platform (AMTI®, model: OR 6-5-1000, Watertown, MA, USA) recorded the ground reaction forces and moments (1 kHz) through dedicated software (Vicon Nexus® 1.7.1). Prior to storage in the computer, the signals were amplified and low-pass filtered at 10 Hz. For the quiet standing sway analyses, center of pressure (CoP) standard deviation, speed, range and CoP mean position in both directions (anterior-posterior and medial-lateral), were calculated based on 50s (first and last 5 seconds were excluded) of the quiet standing tasks. Average scores from three repeated trials were used for analysis.

### Self-reported disease status and pain intensity

All patients completed the Knee Injury and Osteoarthritis Outcome Score (KOOS) [39]. The KOOS is a patient-administered questionnaire with 5 separate subscales assessing pain, quality of life, symptoms, daily living activities and sport and recreation activities. Each item is scored 0–4, and items are summed yielding a total KOOS score and separate subscale scores. The scores are transformed to a 0–100 scale, where 100 represent the best result (i.e., no symptoms). The patients were asked to verbally rate (numeric rating scale) their pain intensities prior to each quiet standing trial as a number between 0 (no pain) and 10 (worst pain imaginable). For the analysis the mean of the scores for pain intensity prior to each trial was used.

### Statistics

For data analysis the KL score from the most affected knee was used to separate the patients in two different groups: (i) “less severe” (KL 1 and 2) and (ii) “severe” (KL 3 and 4).

The data is presented as mean and standard error of the mean (SEM). T-test for independent samples was used to analyze the KOOS data between “less severe” and “severe” groups; non-parametric Mann-Whitney U-test was used for the pain intensity and KL score analyses. A 3-way mixed model analysis of variance (ANOVA) was used to analyze the data, with *disease severity* (less severe, severe) as between-group factor and four sensory conditions implemented as two repeated-measures factors: *surface* (firm and soft) and *vision* (eyes closed and open). Age, gender and body mass index (BMI) were used as covariant variables in the statistical models. In case of significant factors in the ANOVA, Newman-Keuls (NK) post-hoc tests were performed for multiple comparisons. Spearman's rank order correlation analyses were performed between pain intensity, KL score, and the CoP variables that reached significant 3-way interaction in the ANOVA. Significance was accepted at *P*<0.05.

## Results

### Patient characteristics

Forty-two (29 females) of the initial forty five patients were included in the analyses since 3 patients were unable to finish the postural tasks. The remaining patients were classified according to their disease progression [40] (KL score) in (i) “less severe” with KL 1 and 2 (n = 24) and “severe” with KL >2 (n = 18) (Table 1). No significant difference was found between the two groups [mean (± SEM)] in age (“less severe”: 62±6 years and “severe” 63±6 years) or BMI (“less severe”: 35±4 kg*m^−2^ and “severe”: 36±4 kg*m^−2^).

**Table 1:** Characteristics of the KOA patients (n = 42).

Grouping according to KL score		
	Less severe KOA patients (n = 24)	Severe KOA patients (n = 18)
Female/male n°	18/6	11/7
Age (years)	61.2±1.1 (52–74)	64.1±1.7 (50–74)
BMI (kg/m^2^)	35.6±0.8 (30–49)	36.3±0.9 (31–49)
KOOS pain score (0–100)	77±3 (47–94)	64±5 (25–94)
KOOS quality of life score (0–100)	***54±3 (18–87)***	39±5 (6–75)*
KOOS symptoms score (0–100)	***81±3 (54–100)***	62±6 (3–93)*
KOOS daily living activities score (0–100)	77±3 (29–99)	69±4 (35–97)
KOOS sports and recreational activities score (0–100)	41±5 (0–95)	29±5 (0–70)
Pain Intensity immediately before measurements (0–10)	***1.7±0.1 (0–5)***	***2.9±0.7 (0–9)^#^***
KL score (1–4)	***1.8±0.1 (1–2)***	***3.2±0.1 (3–4)^a^***

The knee osteoarthritis (KOA) patients were divided in two groups according to: (i) KL grade: Less severe (KL 1 and 2) and severe (KL >2). Mean ± SEM (Range) for body mass index (BMI), KOOS (Knee injury and Osteoarthritis Outcome Score) scores for pain, quality of life, symptoms and daily living, and pain intensity immediately before the balance measurements and Kellgren and Lawrence (KL) score are presented. Significant group differences are indicated by “*” (t-test) and “^#^” (Mann-Whitney U-Test).^a^ This parameter is different as it is used to separate the groups.

### Severity scores

The average pain intensity was higher in the “severe” patients when compared with “less severe” patients (Table 1). Correlation analyses indicated a positive correlation (R = 0.4) between the degree of the disease progression (KL score) and the average pain intensity reported immediately before the balance trials (Table 2). Compared with the severe group, the less severe group showed significantly better KOOS scores for pain, quality of life, and symptoms (Table 1).

**Table 2:** Correlation between KL scores, Pain Intensity, and CoP Range in the medial-lateral direction during quiet standing (n = 42).

	Spearman R		p-values
KL score vs. Pain Intensity	***0.40***		0.01*
*KL score vs. CoP Range ML*	Spearman R		p-level
Eyes Open – Firm Surface	0.06		0.72
Eyes Closed – Firm Surface	0.01		0.95
Eyes Open – Soft Surface	0.09		0.57
Eyes Closed – Soft Surface	0.14		0.39
*Pain intensity score vs. CoP Range ML*	Spearman R		p-level
Eyes Open – Firm Surface	0.28		0.07
Eyes Closed – Firm Surface	***0.62***		0.01*
Eyes Open – Soft Surface	0.18		0.26
Eyes Closed – Soft Surface	0.18		0.26

Spearman (R) correlation coefficient between pain intensity immediately before each balance measurement, Kellgren and Lawrence (KL) score, Centre of Pressure (CoP) Range in medial-lateral (ML) during four different sensory conditions: (i) Eyes open and firm surface (ii) eyes closed and firm surface, (iii) eyes open and soft surface, and (iv) eyes closed and soft surface. Significant correlations are indicated by *p-values* smaller than 0.05 (*).

### Centre of pressure

**Figure 1:**
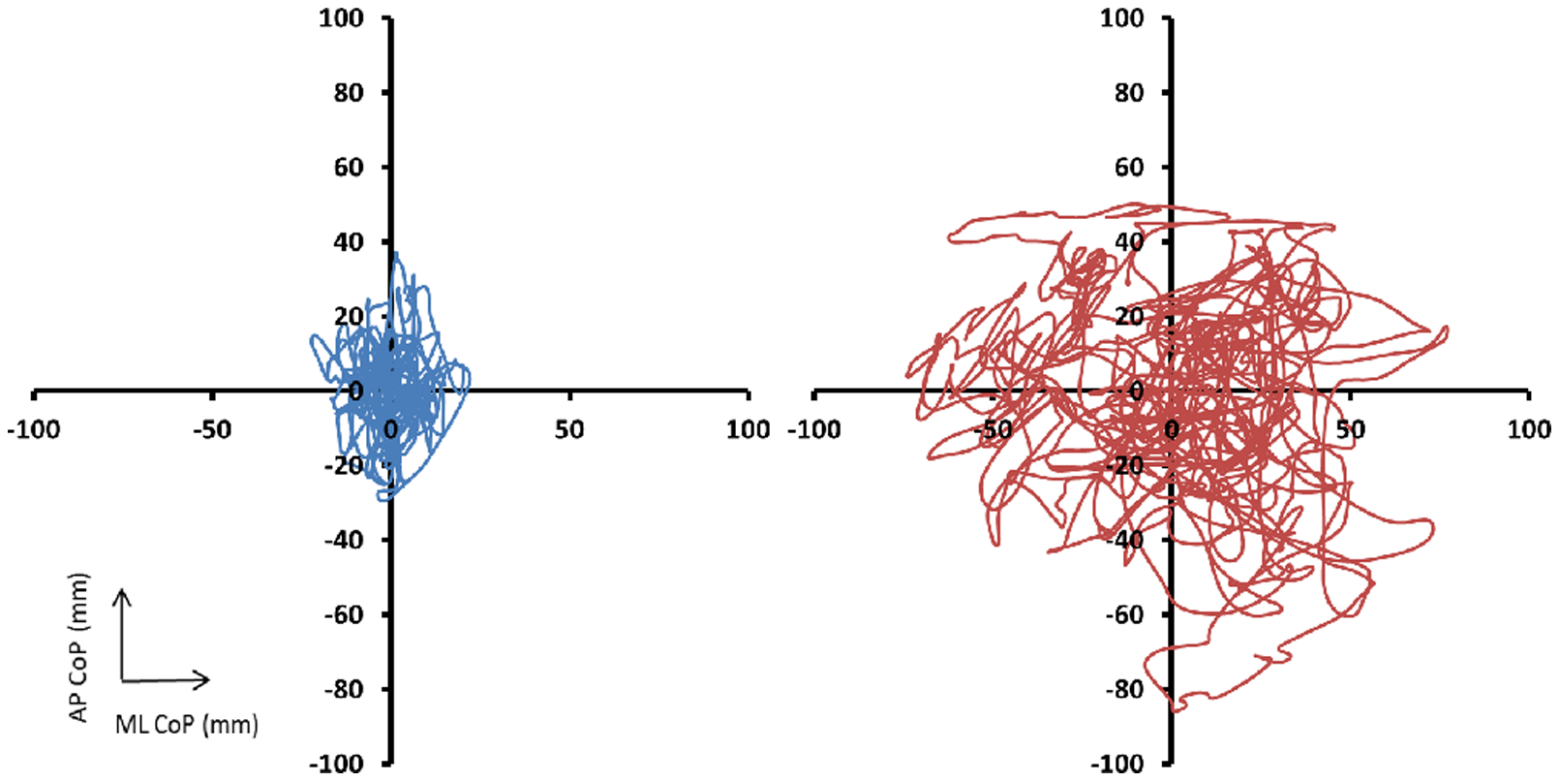
Representative examples of bidimensional center of pressure (CoP) trajectory for a representative subject from the “severe” (red line) and “less severe” (blue line) group during one minute of quiet standing with eyes closed on the soft (foam) surface.

**Figure 2:**
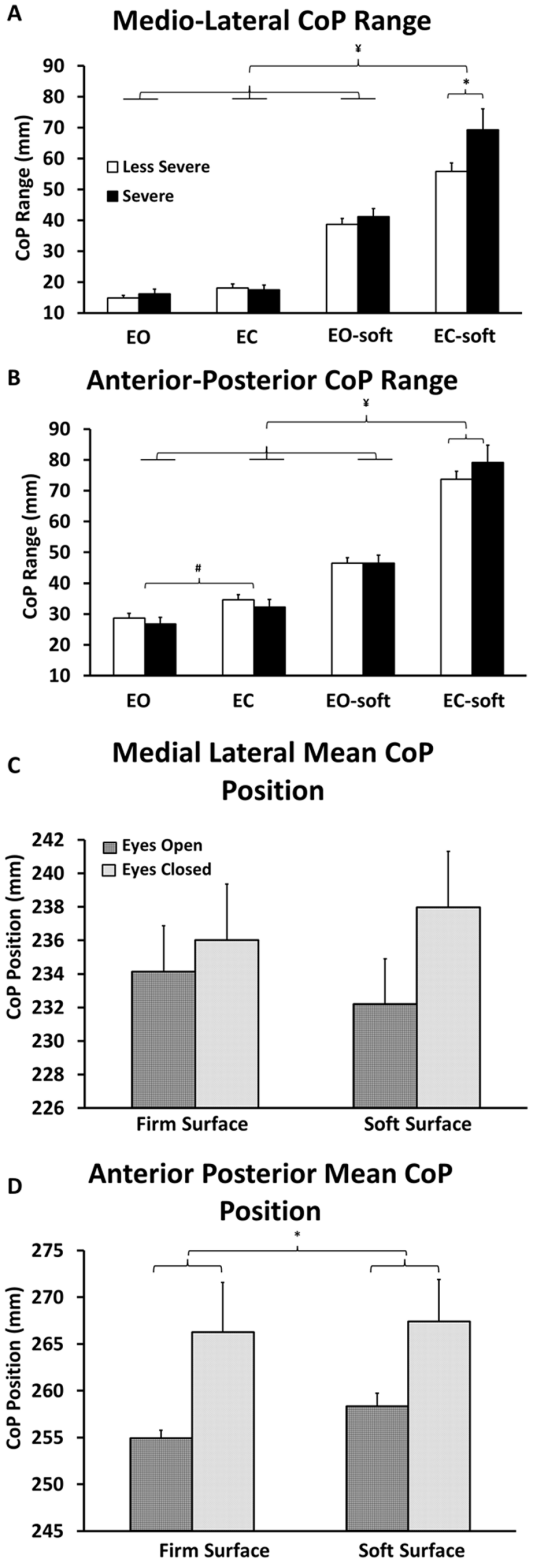
Mean (+ SEM, N = 42) center of pressure (CoP) variables for the “severe” and “less severe” groups during one minute of quiet standing in four different sensory conditions: Eyes open with firm surface (EO), eyes closed with firm surface (EC), eyes open with soft (foam) surface (EO-soft), and eyes closed with soft (foam) surface (EC-soft). **A**: CoP range in the medial-lateral direction for severe and less severe groups. Significantly larger CoP range in the medial-lateral direction: (i) for the “severe” group during EC-soft condition compared with the “less severe” group (*, for the triple interaction between *disease severity*, *surface* and *vision*, NK: *P<0.01*) and (ii) during EC-soft condition when compared with all other conditions (¥, for the double interaction between *surface* and *vision* NK: *P<*0.01). B: CoP range in the anterior-posterior direction for severe and less severe group. Significantly larger CoP range in the anterior-posterior direction: (i) during EC-soft condition when compared with all other conditions (¥, for the double interaction between *surface* and *vision* NK: *P<*0.01) and (ii) during the EC condition compared with EO condition (#, for the double interaction between *surface* and *vision* NK: *P<*0.01). **C**: Mean CoP position in the medial-lateral direction during both *vision* (eyes open and eyes closed) and *surface* (firm and soft) conditions. **D**: Mean CoP position in the anterior-posterior direction was located significantly more anterior in the soft foam surface compared with firm surface regardless the *vision* condition (*, for the double interaction between *surface* and *vision* NK: P<0.01).

Examples of CoP excursion for representative “severe” and “less severe” patients are illustrated in Figure 1. The data for the medial-lateral and anterior-posterior standard deviation and speed is shown in Table 3. In Figure 2, data for CoP range in both medial-lateral (A) and anterior posterior (B) direction and mean CoP positions (C: medial-lateral and D: anterior-posterior direction) are presented. The covariant means for age and BMI were 62.5 years and 35.9 kg*m^−2^ respectively.

**Table 3:** Center of Pressure parameters during quiet standing.

*Centre of Pressure SD – AP (mm)*	EO	EC	EO-soft	EC-soft	p-value
Less Severe	5.4±0.3	6.0±0.3	7.7±0.3	12.2±0.5	<0.01*^,¥^
Severe	5.1±0.3	5.9±0.4	8.2±0.3	14.6±1.5	
*Centre of Pressure SD – ML (mm)*					
Less Severe	2.7±0.1	3.2±0.3	6.8±0.4	9.4±0.5	0.24
Severe	2.9±0.2	3.2±0.2	7.2±0.4	11.4±1.1	
*Centre of Pressure Speed – AP (mm/s)*	EO	EC	EO-soft	EC-soft	
Less Severe	9.4±0.8	13.3±1.4	20.6±2.1	38.6±3.8	<0.01*^,#,¥^
Severe	8.5±1.3	11.1±1.8	20.1±3.3	38.3±6.8	
*Centre of Pressure Speed – ML (mm/s)*					
Less Severe	4.9±0.4	6.2±0.7	14.6±1.5	24.8±2.6	<0.01*^,¥^
Severe	5.0±0.8	5.5±0.9	13.6±2.4	26.9±5.4	

Mean (± SEM) center of pressure parameters from the quiet standing posture during four sensory condition: eyes open and firm surface (EO), eyes closed and firm surface (EC), eyes open and soft surface (EO-soft) and eyes closed and soft surface (EC-soft). The CoP Standard Deviation (SD) and speed in medial–lateral (ML) and anterior–posterior (AP) directions are presented for the less severe (KL score 1 and 2), severe (KL score >2) groups. Significant increases in body sway during EC-soft compared with EO-soft condition are indicated (*, interaction between *surface* and *vision*; NK: *P<*0.01). Significant increase in body sway during EC compare EO condition is marked with “^#^” (interaction between *surface* and *vision*; NK: *P = *0.02). Significant higher values during EC-soft compared with all other conditions (^¥^, Interaction between *surface* and *vision*; NK: *P<0.01*

The ANOVA results indicated a 2-way interaction between surface and vision, where CoP speed (Table 3, ANOVA: F(1,40) = 57.6, P<0.01, NK: P = 0.02) and CoP range (Figure 2B, ANOVA: F(1,40)  = 79.9, P<0.01, NK: P<0.01), both on the anterior posterior direction, were increased during eyes closed condition compared with the eyes open condition when standing on firm surface. In both groups, the mean CoP position in the anterior-posterior direction during soft surface condition was located more anteriorly when compared with the firm surface condition, regardless of visual information available (Figure 2D, F(1, 40)  = 5.1, P<0.03, NK: P<0.01).

Significant 2-way interaction between surface and vision indicated that eyes closed conditions compared with eyes open conditions (while standing on the foam surface), significantly: (i) increased the CoP standard deviation in the anterior-posterior direction (Table 3, ANOVA: F(1, 40)  = 53.3, P< 0.01, NK: P<0.01), (ii) increased CoP speed in the medial-lateral (Table 3, ANOVA: F(1,40) = 42.2, P<0.01, NK: P<0.01) and anterior posterior direction (Table 3, ANOVA: F(1,40)  = 57.6, P<0.01, NK: P<0.01), and (iii) enlarged CoP range in the medial-lateral (Figure 2A, ANOVA: F(1,40)  = 61.9, P<0.01, NK: P<0.01) and anterior posterior direction (Figure 2B, ANOVA: F(1,40)  = 79.9, P<0.01, NK: P<0.01).

Additionally, interactions between surface and vision indicated that during eyes closed condition with foam surface, the body sway was higher than all other conditions for: (i) CoP standard deviation in the anterior-posterior direction (ANOVA: F(1, 40) = 53.3, P<0.01, NK: P<0.01), (ii) CoP velocity in the medial-lateral (ANOVA: F(1, 40) = 42.2, P<0.01, NK: P<0.01) and the anterior-postural direction (ANOVA: F(1, 40) = 57.6, P<0.01, NK: P<0.01), and (iii) CoP range in the medial-lateral (ANOVA: F(1, 40) = 61.9, P<0.01, NK: P<0.01) and anterior-posterior direction (ANOVA: F(1, 40) = 79.9, P<0.01, NK: P<0.01).

A significant 3-way interaction between disease severity, surface and vision indicated that the “severe” group showed significantly larger CoP range in the medial-lateral direction compared with the “less severe” group during the eyes closed with soft surface condition (Figure 2A, ANOVA: F(1, 38)  = 4.9, P<0.03, NK: P<0.01).

#### Correlation between CoP range in the medial-lateral direction, Pain Intensity and KL scores

Correlation analyses using pain intensity and KL scores and CoP range in the medial-lateral direction are presented in Table 2. Pain intensity was positively correlated (R  = 0.62) with CoP Range in the medial-lateral direction during eyes closed condition on firm surface.

## Discussion

The present study assesses different sensory conditions to test how structural disease severity (such as joint narrowing and the presence of osteophytes) at the knee joint and pain intensity affects postural sway. The severe group presented higher levels of pain immediately before the balance tests, worst scores for quality of life and symptoms and increased sway in the medial-lateral direction during eyes closed condition on soft surface when compared with less severe patients. A positive correlation between pain intensity and KL scores was found. Pain intensity was positively correlated with increased postural sway (CoP range in the medial-lateral direction) while standing on firm surface when visual input is not available.

### Postural stability in KOA patients

The relation between knee osteoarthritis severity and postural sway under different sensory conditions is a novel finding. Previous studies on postural stability in KOA patients have mainly focused on the difference between patients and matched control subjects and found that KOA patients have an unstable balance [4], [5], [16]. When the balance of KOA patients on firm surfaces was compared with normal subjects (without symptoms and structural changes), Masui *et* *al.* [17] showed with multiple linear regression analyses that morphological bone changes, such as joint space narrowing and presence of osteophytes in the knee joint, was a significant factor for increased postural sway. In extension of these previous findings, the present study evaluated patients with a range of pain symptoms and structural changes. The results suggest increased postural sway (velocity and range in the anterior posterior direction) on firm surface for both “severe” and “less severe” KOA patients in absence of visual information compared with the open eyes condition. When patients were standing on the soft foam surface, the absence of visual information increased the CoP standard deviation in the anterior-posterior direction and velocity and range in the anterior-posterior and medial-lateral direction. These results suggest that visual information in obese KOA patients plays an important role for balance control, although the disease stages cannot be associated with worsening of balance in the conditions tested in this experiment. As expected, when two senses were manipulated at the same time, postural stability in the obese KOA patients was highly affected (21, 22). Similar results were found in healthy individuals, where simultaneous manipulation of visual and feet proprioceptive information (soft foam surface) increased postural sway range in both young and elderly individuals [41]. In the condition from the present study where the visual information was absent and the feet sole information altered, CoP standard deviation in the anterior-posterior direction and CoP speed and range in the medial-lateral and anterior-posterior direction were increased compared with all other sensory conditions. This result suggests that visual information cannot completely compensate for the lack of information from the feet. Since larger body sway in the medial-lateral direction might be associated with increased risk of falls in elderly [42], increased body sway in both directions suggest that KOA patients are in greater risk of fall when the surface and visual information are not ideal. Alternatively, increased body sway maybe an optimal response for reaching the sensory threshold response in case of degraded sensory inputs [43], [44]. In other words, by increasing body sway, subjects may facilitate their perception of occurring sensory modifications.

Shifting the CoP position forward in the anterior-posterior direction is a normal postural strategy to avoid loss of balance in healthy subjects in presence of experimental knee pain [8]. Similar strategy was also observed in the KOA patients of this study during the soft foam surface compared with firm surface conditions (Figure 2D). This strategy enlarges the distance between the CoP and the posterior boundaries of the base of support [8], [45], and at the same time, it also decreases the distance between the CoP and the anterior boundaries of the base of support. Altogether, this strategy increases the likelihood of reactive strategies, such as stepping forward, to avoid falls when the body is perturbed forward [46], indicating a necessity of KOA patients to prioritize stability against backward perturbations.

### Increased postural sway in severe KOA patients

While standing on soft surface with eyes closed, the severe group swayed approximately 1.5 cm more (CoP range in the medial-lateral direction) when compared to the less severe group (Figure 2A). In this condition, visual information is suppressed and the proprioceptive information from the feet is altered. Therefore, the patients must rely primarily on the vestibular and proprioceptive information from other parts of the body, such as knee and hip and its respective soft tissues (muscles, ligaments etc.). In particular, the knee joint has previously been shown as an important structure in quiet standing control in healthy subjects during normal conditions [47], during experimental pain around the knee joint [8], [9], and in KOA patients [16], [48], [49]. During quiet standing, movements around the knee joint are significantly smaller than the movement involving the ankle joint [18]. Moreover, immobilization of the knee joints increased the body sway of healthy individuals compared with control conditions [47]. Furthermore, painful stimulation in the knee area also increased the body sway in healthy subjects compared with pain free conditions [8], [9]. Previous studies including KOA patients reporting pain, demonstrated that the proprioceptive acuity of the knee joint in this population is impaired which may explain the worsened postural control compared with healthy subjects [16], [48], [49].

### Sensory reweighting in KOA patients

It is crucial that patients are able to reorganize their dependence on each sensory information (visual, somatosensory and vestibular) according to changes in the environment [50]. The sensory reweighting hypothesis suggests that when controlling balance, the central nervous system can dynamically adjust the relative contribution (gain) of each sensory input involved, according to the contextual changes in the environment and therefore, modify postural responses [22]. Conflicting information and abrupt changes in the environment (such as moving across irregular/unstable surface, suffering external perturbations, visual conflict, etc.) poses challenges to the central nervous system. Time is necessary to solve these challenges and execute a proper movement. The more challenging the sensory environment, the longer is the processing time used by the central nervous system to interpret the sensory input available [51], to solve the conflict [52], and to generate the desirable muscular commands to maintain the posture. Therefore, inaccuracy in any of these processing steps may destabilize the posture [24]. For example, computational models indicates that healthy subjects rely mainly on somatosensory information (70%) to control their balance during quiet standing, using only 10% and 20% of visual and vestibular information respectively [53]. Experimental data with healthy subjects also indicated that somatosensory information from the lower limb is the most important information to posture control [54]. Although the contribution of each sensory information in KOA patients are unknown, in presence of moderate levels of pain, the visual information seems to increase its contribution to balance control since positive correlations between pain intensity and postural sway (CoP Range in the medial-lateral direction, Table 2) were found during firm surface conditions with eyes closed. These larger oscillations are indicators that, the higher the pain intensity, the less efficient is the postural control applied to restore an equilibrium position while standing. This lack of effectiveness make the patients more prone to fall if immediate actions fail to restore the balance [50].

Teasdale *et* *al* [41] showed increased postural sway in healthy elderly, compared with healthy young, only when visual and the feet proprioceptive information were manipulate simultaneously. In the present study, it was only possible to differentiate between both KOA groups with regard to their postural sway when dramatic changes (concurrently visual and proprioceptive) were made to the environmental context. In these challenging conditions, adaptive strategies of reweighting the gain of the different sensory inputs are crucial for estimating body dynamics [55] and therefore to maintain balance. Vision is an important source of information in elderly people when maintaining balance [22], [41], [56]. Likewise, the increased body sway in the “severe” patients compared with the “less severe” during eyes closed condition with soft foam surface indicates that “severe” patients relies more in the visual information then the “less severe” patients. Another important contributor for estimating the body dynamics is the knee joint, since it has a large contribution in the proprioceptive information available during altered postural conditions [47]. Previous data showed that structural damage of the knee joint in KOA patients impaired the knee position sense [57] potentially due to altered proprioceptive information from the knee. This may also explain why “severe” patients in this study (with worst knee cartilage damage) showed larger postural sway in the medial-lateral direction when compared with “less severe” patients. Similar increase in postural sway in the medial-lateral direction was reported previously in KOA patients [16] and referred as the best biomarker for falls prediction in elderly people [42]. This probably indicates that the lack or severely altered proprioceptive information from the impaired knees increased the likelihood of a fall accident in the severe KOA patients compared with less severe patients. Experimental pain in healthy subjects also indicated that the closer the painful area is to the knee joint, the worst is the balance control during quiet standing, indicating that pain around the knee joint impair balance, with main impairments in the medial-lateral direction [7]–[9]. There are indications that healthy obese, when compared with normal weight subjects, have increased postural sway, which becomes more evident with absence of visual information [25]. In the present study it is not possible to investigate the role of the BMI in the patient's postural control since all patients in this study were obese (less obese patient had BMI equal to 30.1). However, a previous study indicates that higher BMI is associated with poor balance in KOA patients [32]. Experimental knee pain suggests that in healthy subjects the non-painful knee can provide important information to overcome the sensory impairments from the painful areas [8]. If this interpretation can be translated to the patients in this study, the less affected knee might have provided valuable information for maintaining balance, although the exactly extend of this contribution cannot be retrieved from the present data. The present results suggest that obese KOA patients with moderate levels of pain and severe disease progression around the knee are less capable of re-weighting the remaining sensory information and therefore are at higher risks of losing balance when the environment surrounding poses postural challenges. Another possibility is that the lack of information from the knee joint is larger in the “severe” compared with the “less severe” patients. With less information available, the postural sway de facto increases, which may not be an inability of the CNS to reorganize the sensory information but rather incapacity to compensate for the absence or severely altered proprioceptive information from the impaired knees.

### Conclusions

The results support the hypothesis that, the postural reorganization under manipulation of the different sensory information is deteriorated in KOA patients with severe degeneration and/or high pain intensity when compared with less impaired patients. Vision information seems to account for the postural corrections in conditions where the sensory information from the feet's sole is affected. During these conditions, pain intensity was positively correlated with balance impairments, revealing an important role of pain in postural adjustments of obese KOA patients when the environment poses sensory challenges. There are indications that, in healthy subjects, the dynamic sensory reweighting can be optimized via specific training program and enhance balance in altered environments [58]. Therefore, rehabilitation procedures aiming to train sensory reorganization processes, optimizing the time used to solve the sensory conflict and the motor response and decrease pain, may lead to improvements in balance and reduce risk of falls in obese KOA patients.
